# Generating Novel Brain Morphology by Deforming Learned Templates

**Published:** 2025-03-07

**Authors:** Alan Q. Wang, Fangrui Huang, Bailey Trang, Wei Peng, Mohammad Abbasi, Kilian Pohl, Mert Sabuncu, Ehsan Adeli

**Affiliations:** 1 Stanford University, Stanford, CA 94305, USA; 2 Cornell Tech and Weill Cornell Medicine, New York, NY 10044, USA

**Keywords:** MRI Generation, Morphology, Deformable Templates

## Abstract

Designing generative models for 3D structural brain MRI that synthesize morphologically-plausible and attribute-specific (e.g., age, sex, disease state) samples is an active area of research. Existing approaches based on frameworks like GANs or diffusion models synthesize the image directly, which may limit their ability to capture intricate morphological details. In this work, we propose a 3D brain MRI generation method based on state-of-the-art latent diffusion models (LDMs), called MorphLDM, that generates novel images by applying synthesized deformation fields to a learned template. Instead of using a reconstruction-based autoencoder (as in a typical LDM), our encoder outputs a latent embedding derived from both an image and a learned template that is itself the output of a template decoder; this latent is passed to a deformation field decoder, whose output is applied to the learned template. A registration loss is minimized between the original image and the deformed template with respect to the encoder and both decoders. Empirically, our approach outperforms generative baselines on metrics spanning image diversity, adherence with respect to input conditions, and voxel-based morphometry.

Our code is available at https://github.com/alanqrwang/morphldm.

## 1 Introduction

Morphology is an important characteristic of neuroimaging, known to be related to attributes and/or phenotypes such as age, sex, and disease state [20,31]. A growing literature aims to design generative models for structural brain magnetic resonance imaging (MRI), which synthesize realistic morphology and are specific to conditioned attributes such as age and sex [21,23]. These models offer the potential to augment limited training data [24], generate counterfactuals [15], and increase interpretability [30].

In computer vision, generative models have found success in natural image domains, driven largely by GANs [8,10,35] and diffusion models [13,26]. These models work by learning the data distribution p(x) from which the data is assumed to be sampled. Although highly general and capable of capturing the variety inherent in naturalistic images, this approach applied to neuroimaging may fail to capture intricacies and subtleties crucial to morphology, especially in the context of high-dimensional 3D volumetric data and data-limited scenarios.

Specific to neuroimaging, previous work has shown that much of the variability between individual anatomies can be captured by geometric and topological changes [4,5]. This motivates a line of work that, instead of directly synthesizing images, synthesizes dense deformation fields that are applied to a template image. However, in addition to using less powerful generative models like GANs, prior work either precomputes deformation fields using an expensive registration method [3,37] or fixes the template, thus biasing and limiting the model’s ability to adequately capture the image distribution [3,17,36].

In this work, we propose a 3D brain MRI generation method based on state-of-the-art latent diffusion models (LDMs), called MorphLDM, that generates novel images by applying synthesized deformation fields to a *learned* template. MorphLDM differs from LDMs in the design of the encoder/decoder. First, a learned template is outputted by a template decoder, optionally conditioned on image-level attributes. Then, an encoder takes in both an image and the template and outputs a latent embedding; this latent is passed to a deformation field decoder, whose output deformation field is applied to the template. Finally, a registration loss is minimized between the original image and the deformed template with respect to the encoder and both decoders. Subsequently, a diffusion model is trained on these learned latent embeddings.

To synthesize an image, MorphLDM generates a novel latent in the same way as standard LDMs. The decoder maps this latent to its corresponding deformation field, which is subsequently applied to the learned template. We find that synthetic samples generated by our approach not only outperform powerful baselines on standard image generation metrics like FID, but also better capture input conditions known to be related to morphology, including age and sex. Additionally, we perform a voxel-based morphometry [33] analysis and find that our models generate more realistic structures in terms of regional volume compared to baselines.

To the best of our knowledge, MorphLDM is the first MRI diffusion model that synthesizes deformation fields applied to a learned template. Our approach is efficient and data-driven in the sense that both the deformation fields and the template are learned simultaneously, without requiring precomputation of deformation fields or making a specific choice for the template.

## 2 Background

### 2.1 Brain MRI Generation

Generative adversarial networks (GANs) and their extensions have been widely explored in the context of brain MRIs, including using Wasserstein GANs [11], incorporating variational autoencoders (VAEs) and code discriminators [18], and leveraging segmentations [8]. Autoregressive (AR) approaches generate samples in an autoregressive fashion and require discrete tokenization of images, typically done with a vector-quantized (VQ)-VAE [7,28].

More recently, latent diffusion models (LDMs) have become the state-of-the-art generative model for brain MRIs [21,23], and are trained in two steps. In the first stage, an autoencoder D(E(x))=D(z) is trained on a reconstruction loss $L_{sim}$ to learn a latent space. In the second stage, a diffusion model is trained on the latent space defined by z=E(x); specifically, a network $\epsilon_{\omega }$ is trained to perform time-conditioned denoising of the latent embeddings:

(1) $$\underset{\omega }{\text{argmin}}\;E_{E(x),\epsilon ∼N(0,1),t\in [T]}{\left\|\epsilon -\epsilon_{\omega }\left(z_{t},t,c\right)\right\|}_{2}^{2},$$

where E is expected value, N(0,I) is the standard normal distribution, and c is any conditioning information associated with the image.^3^

### 2.2 Templates

Deformable templates, or atlases, are common in computational anatomy. Research is dedicated to constructing unbiased templates such that the deformation fields from these templates to individual images can be analyzed to understand population variability [2,4,9]. More recently, template learning approaches use neural networks to learn templates that are data-driven and attribute-specific [5,6,27]. These approaches aim to build decoders that learn templates conditioned on specified attributes. In this work, we take inspiration from template learning approaches, where our goal is to learn a universal template concurrently with a deformation field synthesizer to generate novel brain images.

## 3 Methods

Let $x\in {\mathbb{R}}^{C\times L\times W\times H}$ denote a 3D volume sampled from a distribution p(x). For MRIs, x is a single-channel intensity image where C=1. Let z∼p(z) denote a sample from some prior distribution (usually a standard normal) and let c∈C denote (optional) conditioning information. Typical generative approaches aim to model p(x) directly via a generator G that takes in a latent sample along with the condition: $\hat{x}=G(z,c)$.

We assume the existence of a template $\bar{x}\in {\mathbb{R}}^{C\times L\times W\times H}$ such that any x∼p(x) is related to $\bar{x}$ via applying a deformation field $v\in {\mathbb{R}}^{3\times L\times W\times H}:x=v\circ \bar{x}$. Under the template assumption, we can recast modeling p(x) as modeling p(v), the distribution of deformation fields induced by $\bar{x}$. Then, the output of the generator is a novel deformation field $\hat{v}=G(z,\bar{x},c)$, and the novel image derived from this deformation field is $\hat{x}=\hat{v}\circ \bar{x}$.^4^

To prevent any bias due to a specific choice of $\bar{x}$, we learn $\bar{x}$ concurrently with G by optimizing a separate template decoder $\bar{x}:C\to {\mathbb{R}}^{C\times L\times W\times H}$, which outputs a deterministic, universal template on which all synthesized deformation fields are applied. In the simplest case, $\bar{x}$ can be made unconditional by setting the input vector to be learnable. We also experiment with adding c as an input to the template decoder to learn conditional templates. This enables the learned templates to capture intensity or attribute-specific structural detail that can improve overall generation [5,6].

### 3.1 MorphLDM

**Fig. 1.**
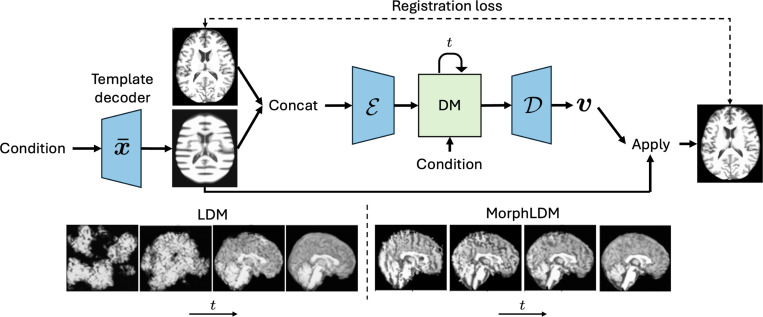
(top) Graphical depiction of MorphLDM. The encoder takes in an image and a template that is the output of a template decoder, concatenated along the channel dimension. The decoder outputs a deformation field v that is applied to the template. Encoder-decoder networks in blue are trained in the first stage on a registration loss (similar to LDM). The diffusion model (DM) in green is trained on the learned latents in the second stage. (bottom) Visualization of denoising steps t∈[T] of LDM (left) and MorphLDM (right). While LDM’s decoder maps from a synthesized latent to an image directly, MorphLDM’s decoder maps to a synthesized deformation field, which is subsequently applied to the learned template.

In this work, we apply our approach to state-of-the-art LDMs, where the second stage diffusion training remains unchanged (Eq. (1)) and the main changes are in the design and training of the autoencoder. Fig. 1 gives a graphical depiction. Let $E_{\theta }$ and $D_{\phi }$ denote the image encoder and decoder parameterized by θ and ϕ, respectively. The input to the autoencoder is x and $\bar{x}$ concatenated along the channel dimension. The encoder output z is passed to the decoder, which outputs a deformation field v that is applied to the template $\bar{x}$ via a differentiable grid-sampler. Thus, the objective is

(2) $$\underset{\bar{x},\theta ,\phi }{\text{argmin}}\;E_{x}\left[L_{sim}\left(D_{\phi }\left(E_{\theta }(x,\bar{x})\right)\circ \bar{x},x\right)+R(v)\right],$$

where R(v) denotes a regularization term on the predicted deformation field. In this work, we penalize the magnitude and spatial gradient of the displacement field. Letting $u_{v}$ denote the spatial displacement for $v=Id+u_{v}$, where Id is the identity transformation: $R(v)=\alpha {\left\|u_{v}\right\|}_{2}+\beta {\left\|\nabla u_{v}\right\|}_{2}$. Following prior work [23,26], we also impose a slight KL penalty towards a standard normal on the latent space to prevent arbitrarily high-variance latent spaces.^5^.

## 4 Experiments

### Dataset.

We train all models on a large dataset of publicly-available T1-weighted brain MRIs.^6^ For all datasets, we restrict to healthy controls when applicable. In total, we have 27,066 3D volumes, of which we reserve 21,051 for training and the rest for validation. Each volume is of resolution 160×192×176 and skullstripped and registered to MNI space. Age and sex metadata is available for all images. Since our dataset is skewed toward younger ages, we uniformly sample ages binned across decades during training.

For generative model evaluation, we generate 1000 samples with conditions linearly spaced between the ages of 5 to 100 and sexes are evenly split between male and female. For real samples, we collect 1000 validation samples by collecting 500 female validation samples closest in age to the synthetic samples (without replacement), and similarly for male samples.

### Architectural and Training Details.

For all LDM-based models, the encoder E is composed of convolutional blocks with 3 levels of downsampling; the number of latent channels is 8. For LDM, D outputs a 1-channel reconstruction. For MorphLDM, it outputs a 3-channel output for a 3D deformation field. The diffusion UNet $\epsilon_{\omega }$ has intermediate channel sizes of [384, 512, 512], and cross-attention is applied with c at the latter two levels. The architecture of the template decoder $\bar{x}$ is similar to the decoder for the deformation field, except that it takes as input a vector with a size equal to the number of conditions c and outputs a template $\bar{x}\in {\mathbb{R}}^{C\times L\times W\times H}$.

For training, we use the same reconstruction/similarity loss $L_{sim}$ for all LDM-based models, which is composed of an L1 loss and an adversarial loss using a patch-based discriminator [14] with a weight of 0.005. A loss coefficient of 1e−7 is used for the KL penalty. For MorphLDM, we set α=5 and β=1. All training and evaluation is done on an NVIDIA H100 GPU.

### Baselines.

We compare against two GAN-based models, VAE-GAN and α-GAN [18], and an autoregressive model (AR) trained on discrete tokens learned through a VQVAE [28]. We also compare against an LDM model, which minimizes a reconstruction loss $L_{sim}$ during autoencoder training. All architectural and training details are kept as identical as possible between LDM and MorphLDM variants.

All LDM-based models have conditioning information *c* injected in the diffusion UNet $\epsilon_{\omega }$ via cross-attention with the intermediate feature maps. We also experiment with including c in the autoencoder by concatenating age and sex as additional channels of z repeated across the spatial grid. For MorphLDM, c is also passed as input to the template decoder $\bar{x}$. Throughout this subsection, LDM-based models which have conditioning information injected in the encoder/decoder are denoted with a superscript ^c^.

### Metrics.

We report the Frechet inception distance (FID) [12] using pretrained ImageNet weights, which has been shown to more closely align with expert judgment [34]. To quantify diversity, we report the multi-scale structural similarity measure (MS-SSIM) [23,32], which is computed by averaging over 1000 MS-SSIM values for 1000 pairs of images. Lower is better since a higher MS-SSIM indicates a higher degree of image similarity and therefore, lower diversity.

To measure how well synthetic samples capture age and sex information, we pass the samples through pretrained models for age and sex and quantify the prediction error. We train a CNN-based age regressor and sex classifier on the same (real) data used for training the generative models.^7^

We perform a voxel-based morphometry analysis to evaluate synthetic samples with respect to volumes of brain regions [33]. To obtain segmentations for both real and synthetic data, we use SynthSeg [1], a widely-used tool for segmenting brain MRI volumes.

### 4.1 Image Generation

**Fig. 2.**
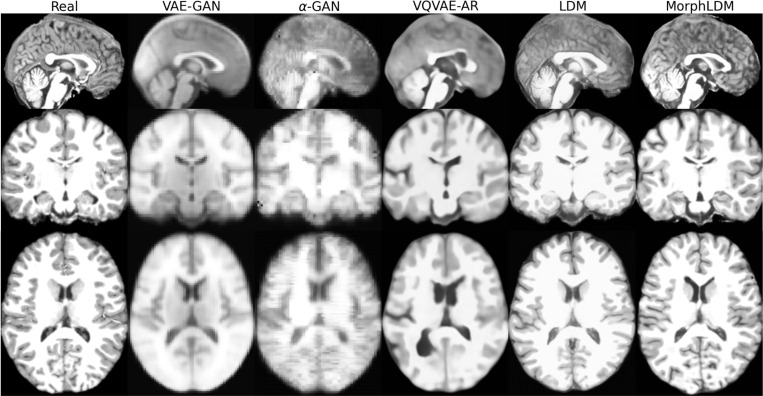
Representative real and synthetic samples for all models shown in the three planes. Age=10, sex=“female”. We observe that MorphLDM has a higher degree of morphological detail compared to baselines, especially in folding patterns in cortical regions.

Fig. 2 depicts a representative sample across three views for all models. We observe that LDM-based samples exhibit sharper images, with GAN-based models producing blurry results as well as less diversity across samples. We observe that MorphLDM has a higher degree of morphological detail compared to LDM, especially in folding patterns in cortical regions. This may be attributed to the model’s ability to focus on morphology instead of other aspects of the image.^8^

Table 1 summarizes the results on various image generation metrics. We find that both MorphLDM variants better capture age and sex information, as evidenced by higher sex accuracy and lower age MAE. MorphLDM^c^ generally outperforms MorphLDM, which can be attributed to its increased expressivity in being able to generate age-conditioned templates. MorphLDM variants also outperform LDM variants on FID and MS-SSIM. We attribute this to MorphLDM’s ability to capture more detailed and varied morphology compared to LDM.

**Fig. 3.**
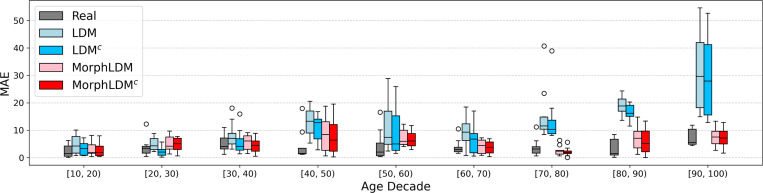
Prediction error of synthetic samples on a pretrained age predictor across age decades. “Real” denotes prediction error on real (i.e. validation) data. Generally, we find that Morph models exhibit lower MAE values than the LDM model across all age ranges, especially at older ages, where less training data is available. MorphLDM^c^ generally outperforms MorphLDM.

Fig. 3 plots the age MAE across age decades for all real and synthetic samples. The improvement of MorphLDM over baseline LDM becomes more pronounced as age increases. Given that our training dataset is skewed toward brain MRIs of younger subjects (5–20 years), we hypothesize that MorphLDM is more data-efficient due to restricting its modeling to morphology.

### 4.2 Effect Size of Regional Volumes

**Fig. 4.**
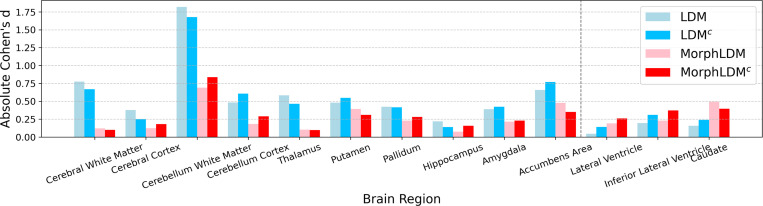
Absolute Cohen’s d of regional volumes between real and synthetic samples. Lower is better. MorphLDM variants exhibit lower values for 10 out of 13 regions.

To quantify the morphological realism of synthetic images, we quantify the effect size of regional volumes between populations of synthetic and real brains [21,19] using the absolute Cohen’s d.^9^ Fig. 4 shows a bar graph of absolute Cohen’s d values for different models, across all brain regions that are segmented by SynthSeg. We find that MorphLDM models exhibit lower Cohen’s d values for 10 out of the 13 regions (left of the dotted line). This indicates a broadly closer degree of anatomical similarity with real samples in terms of regional volumes.

## 5 Conclusion

We propose MorphLDM, a diffusion model for structural brain MRI generation that synthesizes novel displacement fields applied to a learned, universal template. The “autoencoder” of MorphLDM takes as input an image and learnable template and outputs a dense deformation field; this deformation field is applied to the learnable template, and a registration loss is minimized between the original image and the deformed template. Empirically, our approach outperforms baselines with respect to standard image quality metrics, faithfulness to specified attribute conditions, and voxel-based morphometry.

## Figures and Tables

**Fig. 5.**
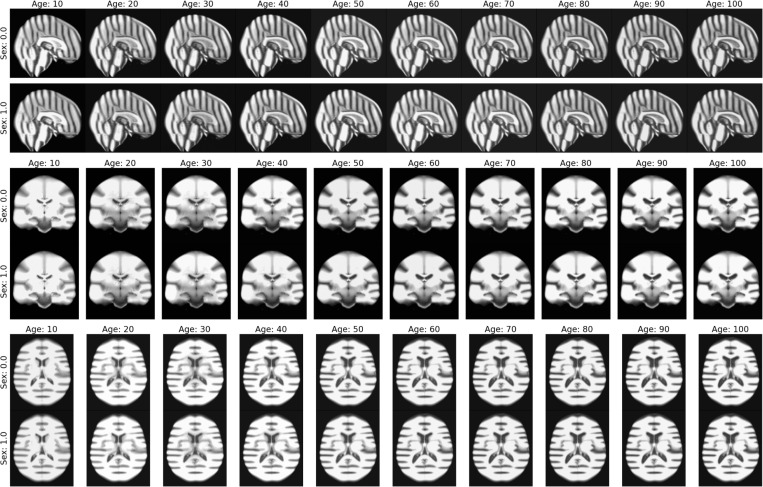
Learned conditional templates for MorphLDM^c^, for varying ages and sexes.

**Fig. 6.**
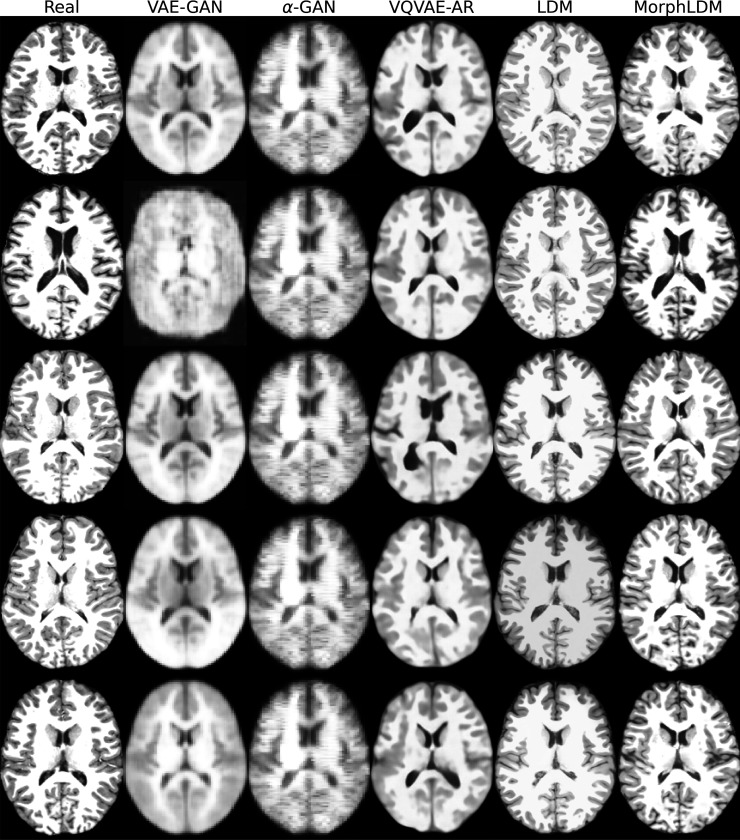
Representative real and synthetic samples.

**Fig. 7.**
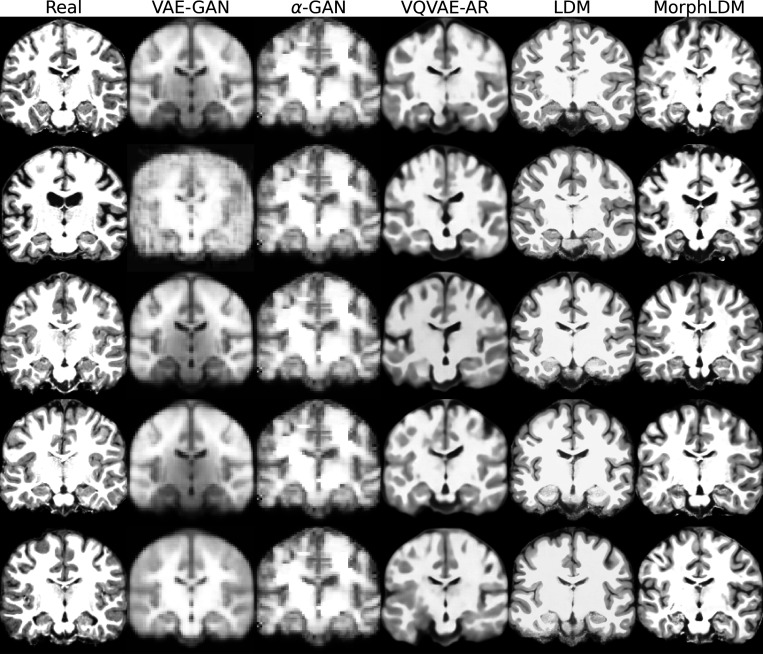
Representative real and synthetic samples.

**Fig. 8.**
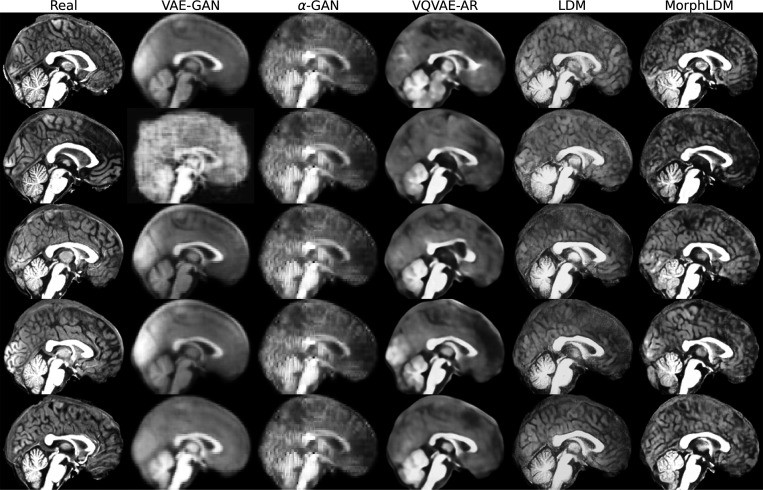
Representative real and synthetic samples.

**Fig. 9.**
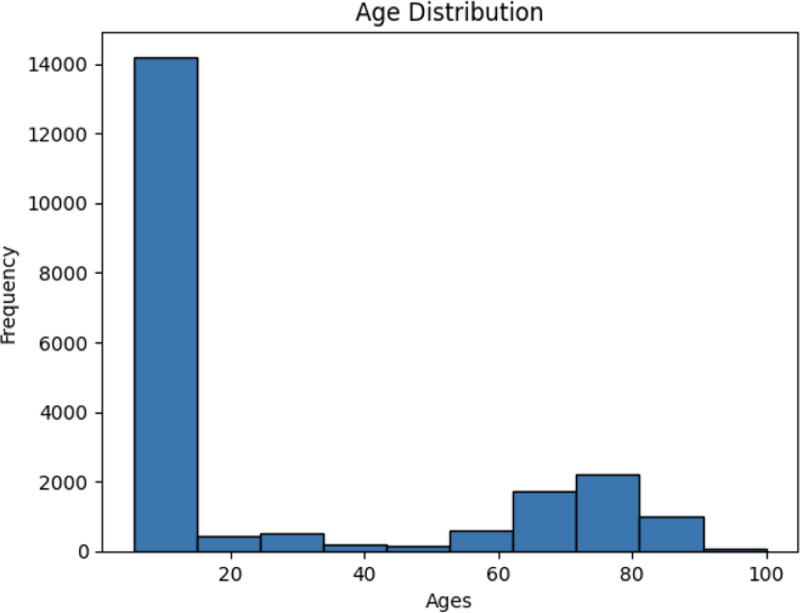
Histogram of age in training dataset.

**Table 1.** Image quality metrics.

	Sex Acc ↑	Age MAE ↓	FID ↓	MS-SSIM ↓
Real	0.88	4.63	–	0.74

VAE-GAN	0.55	35.53	298.38	0.81
α-GAN [18]	0.51	33.30	313.58	0.90
VQVAE-AR [28]	0.62	29.62	290.01	0.80

LDM [23]	0.78	12.95	273.71	0.77
LDM^c^	0.79	10.37	282.52	0.79
MorphLDM (ours)	0.84	5.59	213.70	**0.73**
MorphLDM^c^ (ours)	**0.85**	**4.93**	**202.49**	**0.73**

↑: higher is better, ↓: lower is better

## Footnotes

3 Typically, $\epsilon_{\omega }$ is implemented as a time-conditional UNet [26], where the conditioning information c is included via cross-attention with the intermediate feature maps.

4 The template assumption is common and well-motivated in the literature [3,4,5], but notably may be violated when x exhibits lesions or large structural abnormalities.

5 Many works in this space utilize diffeomorphic constraints [2,5,24]. We found experimentally that this constraint was too strong and led to artifacts in the synthesized samples.

6 Data are from the Alzheimer’s Disease Neuroimaging Initiative (ADNI) [22], Adolescent Brain Cognitive Development (ABCD) Study [16], Human Connectome Project (HCP) [29], and Parkinson’s Progression Markers Initiative (PPMI) [25].

7 The architectures for the age and sex predictors are CNNs with 4 downsampling levels and 2 convolutional blocks (conv, norm, and ReLU) per level. The final layer is a channel-wise average pooling followed by a fully-connected layer down to a scalar output. The age regressor is trained to minimize MSE loss and the sex classifier is trained to minimize binary cross entropy.

8 We refer the reader to the Supplementary material for a visualization of the learned templates for MorphLDM^c^.

9 Absolute Cohen’s d is defined as the absolute difference between the two population means divided by the pooled standard deviation: $d=\frac{\left|{\bar{x}}_{1}-{\bar{x}}_{2}\right|}{s}$.
